# Electrical Response of Different Crystalline Microregions in Poly(vinylidene fluoride)

**DOI:** 10.3390/nano14191555

**Published:** 2024-09-26

**Authors:** Mengyue Su, Jun Zhou, Yuqing Chen, Yilong Wang, Gan Jin, Haiyang Wang, Jiacheng Zhou, Xiaoyue Pang, Zepeng Lv, Kai Wu

**Affiliations:** Center of Nanomaterials for Renewable Energy, State Key Laboratory of Electrical Insulation and Power Equipment, Xi’an Jiaotong University, Xi’an 710049, China

**Keywords:** polyvinylidene fluoride (PVDF), crystalline phase, Kelvin probe force microscopy (KPFM), charge injection, charge dissipation

## Abstract

The crystal structure has a great influence on the dielectric and piezoelectric performance of poly(vinylidene fluoride) (PVDF). In this work, we prepared PVDF films with two typical crystalline phases (α and β). In situ Kelvin probe force microscopy (KPFM) and Piezoelectric force microscopy (PFM) were employed to investigate the responses of different PVDF crystalline phases to charge mobility, polarization, and piezoelectric properties. We used a homemade Kelvin probe force microscope (KPFM) to inject charges into the two crystalline phases to investigate the differences in the response of different crystalline phases of PVDF to electrical excitation on a microscopic scale. It was found that the α-phase has a lower charge injection barrier and is more susceptible to charge injection and that the α-phase is accompanied by a faster charge dissipation rate, which makes it easier to accumulate charge at the interface between the α-phase and β-phase PVDF. Moreover, the PFM polarization manipulation showed no change in the amplitude and phase diagram of the α-phase under ±10 V bias. In contrast, the β-phase showed a clear polarization reversal phenomenon and a significant increase in piezoelectric amplitude, which is consistent with its polar intrinsic properties. This study provides valuable insights into the multiphase contributions and a reference for designing advanced PVDF dielectrics.

## 1. Introduction

The semi-crystalline polymer polyvinylidene fluoride (PVDF), which has unique piezoelectric, high dielectric, and ferroelectric properties, is widely used in the development of sensors, actuators, and energy harvester systems owing to its low price, high flexibility, good biocompatibility, excellent aging resistance, and chemical stability [1,2,3]. The outstanding properties of PVDF are closely related to its crystallinity, crystal structure, and orientation [4,5,6,7]. PVDF exhibits complex crystalline polymorphs, and five crystalline phases of α, β, γ, δ, and ε have been reported [8]. The different crystalline phases of PVDF exhibit different properties. The β phase typically shows the best piezoelectric, ferroelectric, and thermoelectric properties compared to the non-polar α phase [9]. The dielectric and piezoelectric properties of PVDF and the space charge effect at the PVDF-doped interface have been extensively studied [10,11,12,13]. However, due to the lack of high-resolution test equipment and the requirement for microscopic samples, there are still few studies on the electrical properties of PVDF at the nanoscale. There is a lack of studies on the single-crystal charge behavior and piezoelectric properties of the different crystalline phases of PVDF at the nanoscale, so it is difficult to relate the microscopic charge behavior to the molecular chain configurations as well as to the microscopic electrical performances of the PVDF. The electrical properties of different crystalline microregions of PVDF have yet to be investigated, which is crucial for studying the influence of crystal structure on the dielectric and piezoelectric properties of PVDF to fabricate related high-performance functional films and devices.

Previous work has mostly been based on macroscopic characterization methods such as X-ray diffraction (XRD), Fourier transform infrared (FTIR) spectroscopy, differential scanning calorimetry (DSC), and Broadband dielectric impedance spectrometry to study the crystalline phase composition and dielectric properties of PVDF. However, there is a lack of direct characterization of the morphological features and electrical properties of the different crystalline phases at the micro-scale. Atomic force microscopy (AFM) with a nanoscale resolution is a powerful assistant for further characterizing and investigating the micro-regions of material at the micro-scale. AFM provides information on the morphology of the sample surface and a range of in situ electrical, mechanical, and other characteristics of the sample in various extended modes. Piezoelectric force microscopy (PFM) characterizes ferroelectric sample deformation in contact mode and evaluates its properties by measuring the dynamic electromechanical response of the contact surface [14,15]. Ferri et al. utilized PFM to visualize individual piezoelectric BaTiO_3_ nanofillers directly dispersed in a PVDF matrix and monitor the polarization state. This allows for an in-depth study of electroactive composites at the nanoscale [16]. Using PFM, Fortunato et al. investigated the piezoelectric properties of PVDF composite films when nanofillers such as graphene nanoparticles or ZnO nanorods were added to induce the formation of β-phase crystals without applying any electrodeposition [17]. Kelvin probe force microscopy (KPFM) enables the imaging of the surface potential distribution at a high spatial resolution by measuring the local contact potential difference between the tip and the sample [18]. In the application of KPFM, it is even possible to inject charges into the sample micro-region via the conductive tip and analyze the response of the material micro-region under various electrical excitations in situ. There are two important directions in the study of interfacial charge behavior based on KPFM, one of which is the study of the charged nature of the contact charging phenomenon of different materials and the microscopic mechanism of its generation. The second is to study the charge distribution characteristics at the interface formed by the filler particles of composites and matrix materials. Amangeldinova et al. investigated the contact initiation process at the metal–polyvinylidene fluoride (PVDF) interface based on KPFM [19]. Chang et al. observed the surface potential around the charge injection region in Polyethylene/SiO_2_ by using KPFM to investigate the microscopic charge movement properties of the nanodielectric [20]. Moran et al. employed a dynamic KPFM technique to directly monitor the charge dissipation at the nanoscale after locally charging the BaTiO_3_ dielectric film with a conductive tip [21]. Chen et al. used KPFM to analyze the charge injection behavior and charge dissipation behavior at the nano-interface between BaTiO_3_ nanoparticles and the PVDF matrix [22]. However, previous scholars focused on studying the charge behavior between PVDF and doped particles, and at present, people are still puzzled about what kind of charge behavior the different crystalline phases of PVDF experience, so it is necessary to carry out research on the single-crystal charge behavior and piezoelectricity performance of different crystalline phases of PVDF and to make the electrical properties of different crystalline micro-regions of PVDF clear.

Therefore, this work first explores the difference between the α-phase and β-phase responses under the effects of charge injection and bias polarization on a microscopic scale employing the powerful micro-electrical characterization capability of atomic force microscopy (AFM). The influential relationship between the electrical response of different crystalline phases and macroscopic dielectric properties was revealed by the responses of different PVDF crystalline phases to charge mobility, polarization, and piezoelectric properties.

## 2. Experimental Section

### 2.1. Materials

PVDF was supplied by Alfa Aesar Co., Inc. (Shanghai, China). N, N-dimethylformamide (DMF, AR) was purchased from Shanghai Aladdin Biochemical Technology Co., Ltd. (Shanghai, China).

### 2.2. Fabrication of Films

**Figure 1:**
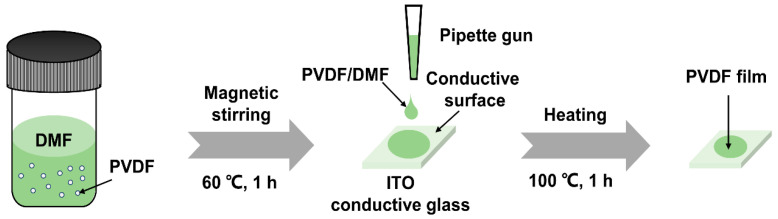
Preparation flowchart of pure PVDF films for microscopic characterization.

Figure 1 shows the process of preparing the sample for microscopic characterization. Firstly, the PVDF powder was first dissolved in the DMF solution at a ratio of 0.0235 g of PVDF/5 mL of DMF and stirred magnetically at 60 °C for 1 h. Then, two drops of the low-concentration solution were transferred to the conductive side of indium tin oxide (ITO) conductive glass using a pipette gun and immediately transferred to an oven preheated at 100 °C and heated for 1 h to prepare a PVDF film with a thickness of about 250 nm.

### 2.3. Characterization

The microstructures of the PVDF films were observed by an Atomic Force Microscope (AFM, Dimension Icon, Bruker, Germany). An X-ray diffractometer (Bruker D8A A25 X, Bruker, Germany) carried out a phase analysis of PVDF films. The Cu-Kα radiation source (λ = 0.154 nm) was used for the test; the test voltage was 20 kV, the 2θ angle ranged from 10° to 40°, and the scan rate was 5°/min. The phase composition of the films for the macroscopic property test was analyzed using an FTIR spectrometer (Nicolet iS10, Thermo Fisher Scientific, Bedford, MA, USA). The test was performed in attenuated total reflectance (ATR) mode with a scanning range of 525~1500 cm^−1^.

### 2.4. Electrical Response

The scanning process for the traditional amplitude-modulated KPFM (AM-KPFM) was divided into two steps. The first step (the main scanning) used the force between the tip and the sample to obtain the surface morphology of the sample. In the second step (the interleave scanning), the probe was lifted to a constant height above the sample surface. At this time, the tip and the sample formed a micro-capacitor system. Due to the difference in their Fermi levels, a surface potential difference (Δ*V*_SP_) between the tip and the sample surface was created. According to the principle of the Kelvin method, the feedback system would additionally compensate for a controllable DC bias (*V*_dc_) within the sample (or tip) to offset the Δ*V*_SP_. To accurately judge whether the potential difference has been completely compensated for, an AC voltage (*V*_ac_) with a frequency of 5000 Hz was also applied to the loop.

**Figure 2:**
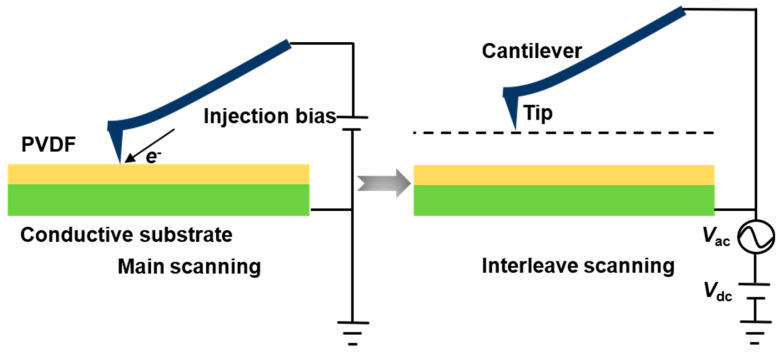
Schematic diagram of the charge injection method based on PeakForce AM-KPFM.

The conductive surface of the ITO glass was connected to the metal round table through a silver conductive paste. The sample to be tested was purged with a deionization air gun to expel any accumulated charges that may be present in the sample. In this work, the PeakForce AM-KPFM developed by Bruker was used to obtain the surface morphology and corresponding mechanical information of the sample by precisely controlling the up-and-down movement of the scanning tube [23]. A bias voltage was applied to the conductive tip during the main scanning to inject charges into the sample surface. The PeakForce AM-KPFM mode can be regarded as a combination of the PeakForce Tapping mode and the AM-KPFM mode, in which two scans are carried out in each measurement; the first scanning of the sample’s surface morphology and mechanical properties in the PeakForce Tapping mode is called the main scan, and the second scanning in the AM-KPFM mode is called the interleaved scan. As shown in Figure 2, AM-KPFM utilizes the tap mode to achieve surface topography imaging of the sample during the main scanning process, while a bias voltage is applied to the conductive tip to inject charge into the sample surface; after charge injection is completed, the probe is lifted to a certain height to perform interleaved scanning to obtain information about the charge left on the sample after the charge injection operation. Since this experiment applied a *V*_ac_ to the sample, the surface potential difference (Δ*V*_SP_) between the tip and the sample was expressed as

(1) $$∆V_{SP}=V_{tip}-V_{sample}$$

where *V*_tip_ is the potential of the tip and *V*_sample_ is the potential of the sample. That is, the larger the potential difference acquired, the lower the potential of the sample. To visually express the effect of the injected charges on the surface potential of the sample, it is noted that the curve data drawn by the extracted data represents the −Δ*V*_SP_ signal, while the potential image obtained by the AFM analysis software (NanoScope Analysis 2.0) represents the Δ*V*_SP_ signal.

### 2.5. PFM Test

The inverse piezoelectric effect of ferroelectric materials was exploited to test the piezoelectric properties of the materials using a piezoelectric force microscope (Dimension Icon, Bruker, Germany). In the PFM test, an AC-driven signal was applied to the tip, which was kept in contact with the sample surface in the scanning. Driven by the AC voltage, the sample expanded and contracted, which caused the tip to generate deflection. The amplitude and phase of the vertical deflection signal were calculated by the component of the tip deflection signal that was in phase with the AC driving voltage and the component that was 90° different from the AC driving voltage, and the required piezoelectric information was obtained.

## 3. Results and Discussion

### 3.1. Morphological and Structural Characterizations

**Figure 3:**
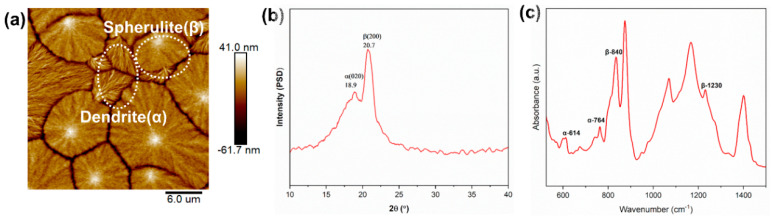
(**a**) Surface microstructural AFM topography images, (**b**) XRD patterns, and (**c**) FTIR spectra images of PVDF films prepared.

Figure 3a shows the observation of two polymer crystals with different morphological characteristics by AFM. The two are characterized by compact spherulite and radial dendrite, respectively. The morphology of the α-phase is dendritic, while the spherical crystals are the β-phase [24]. XRD is an effective method to determine the polycrystalline nature of PVDF. Figure 3b shows the XRD patterns of the PVDF films prepared. The crystallized PVDF film shows diffraction peaks at 2θ = 20.6° and 36.8° originating from the β phase, in addition to the diffraction peak at 2θ = 18.9° corresponding to the (020) crystal plane reflection in the α phase [25,26]. FTIR showed that high-intensity spectral peaks in 876–885, 1067–1075, 1171–1182, and 1398–1404 cm^−1^ occur in the three phases of PVDF [27]. As shown in Figure 3c, the prepared samples have characteristic peaks for both the α phase (614, 764 cm^−1^) and β phase (840 cm^−1^) [28]. Also, a characteristic peak (1230 cm^−1^) representing the β phase appears [29].

### 3.2. Response Differences of PVDF Crystals under Charge Injection

#### 3.2.1. Regional Charge Injection

**Figure 4:**
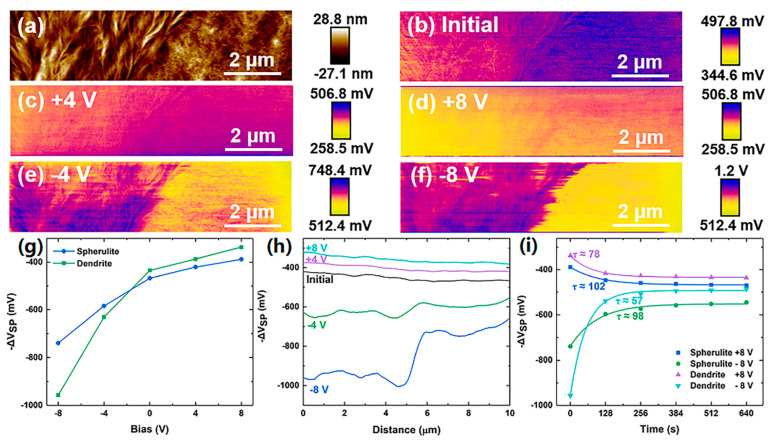
(**a**) Height and (**b**) initial Δ*V*_SP_ images of the regional charge injection region (**c**–**f**) Δ*V*_SP_ images obtained at different biases; (**g**) variation in the average −Δ*V*_SP_ in the dendritic α phase and spherulitic β phase with the magnitude of the tip bias; (**h**) the midline data of − Δ*V*_sp_ extracted from (**b**–**f**); and (**i**) potential decay in the dendritic α phase and spherulitic β phase after the ±8 V charge injection.

During the main scanning, charges were injected into the region with both α- and β-PVDF by applying ±4 and ±8 V tip biases to the conductive tip to investigate the response of different crystalline phases of PVDF to the charges. The spatial distribution of spherulitic and dendritic crystals can be distinguished from the height shown in Figure 4a, with dendrite on the left and spherulite on the right. The Δ*V*_SP_ image in Figure 4b shows that the two crystals’ initial potential states are slightly different. The initial potential state is used as a reference to analyze the changes in the surface potential of the sample under different charge injection biases. Figure 4c–f are the Δ*V*_SP_ images obtained during regional charge injection at ±4 V and ±8 V bias, respectively. When there is a positive DC bias between the tip and the substrate, the Fermi energy of the tip is shifted, and hole injection from the tip to the surface fills more traps on the sample surface [30], resulting in an enhancement in the measured surface potential and a more significant increase in the potential of the dendritic α phase. When there is a negative DC bias between the tip and the substrate, which is not conducive to trap filling, the holes are extracted by the tip, and the overall potential of the sample reduces. Again, the potential of the dendrite reduces more significantly. To more intuitively compare the response differences of PVDF crystals under positive and negative charge injection conditions, the average Δ*V*_SP_ of the dendritic and spherulitic regions and the midline data of the Δ*V*_SP_ images were obtained, and the opposite number of the Δ*V*_SP_ signals were taken to plot in Figure 4g and Figure 4h, respectively. The two figures clearly reflect that the dendritic crystallization is more susceptible to charge injection than the spherulitic crystallization. The potential decay in the dendritic crystallization and spherulitic crystallization after the ±8 V charge injection was also investigated, as shown in Figure 4i. When charges of the same polarity are injected, the potential of the dendrite decays faster than the bulk. When the same polarity charges are injected, the dendrite potential decays faster than the spherulite.

#### 3.2.2. Single-Point Charge Injection

**Figure 5:**
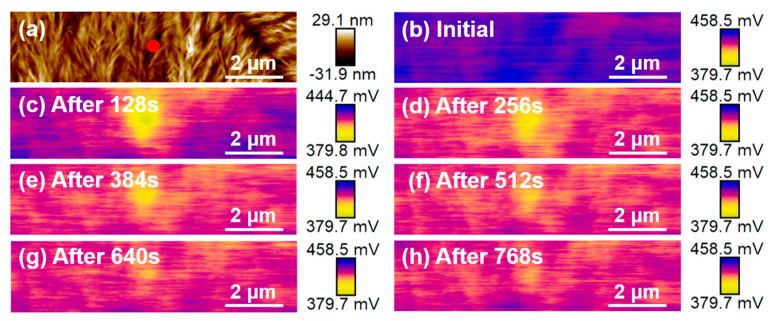
(**a**) Height (the red dot represents the charge injection site) and (**b**–**h**) Δ*V*_SP1_ images of the single-point positive charge injection region of the dendritic α phase.

Notably, ±10 V single-point charge injection was performed at the center of the dendrite and spherulite for 10 min, respectively, and the information about the potential decay after the injection was obtained. As shown in Figure 5c, when the probe tip is in contact with the dendrite, the positive charges are transferred from the tip to the center of the sample, resulting in a brighter area in the center of the Δ*V*_SP_ image. The brighter area means that the potential is lower here, which suggests that the positive charges were successfully injected into the center point of the sample by the tip. In the subsequent Δ*V*_SP_ images, the brighter area at the center gradually disappears. In addition, the height and Δ*V*_SP_ images of the single-point charge injection of −10 V in the dendrite center and ±10 V in the spherulite center are shown in Figures S1–S3, respectively.

**Figure 6:**
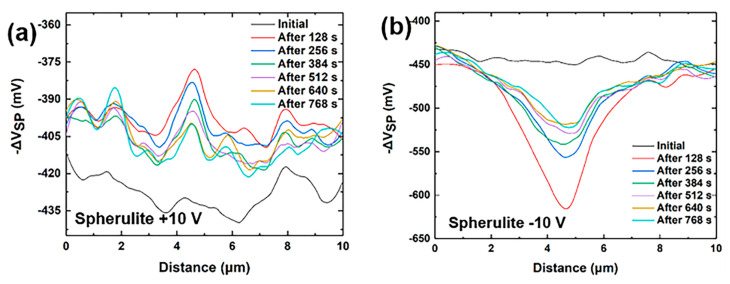

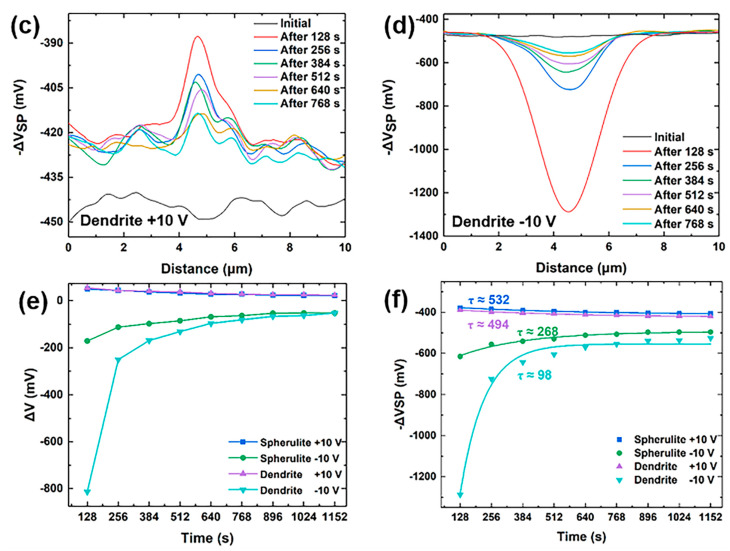
Variation in the midline data of −Δ*V*_SP_ over time after single-point charge injection. The spherulitic β phase injected with +10 V (**a**) and −10 V (**b**), and the dendritic α phase injected with +10 V (**c**) and −10 V (**d**). (**e**) The change in the Δ*V* (the Δ*V*_SP_ at the injection center minus the initial value difference) with time, and (**f**) the potential decay after the single-point charge injection.

To visualize the change in potential at the center over time caused by the charge injection, the midline data of the −Δ*V*_sp_ before and after the charge injection are plotted, as shown in Figure 6a–d. A large value in the curve corresponds to a higher sample surface potential, and a small value corresponds to a lower sample surface potential. The highest potential point can be seen at the charge injection center after the positive charge injection. The lowest potential point appears at the charge injection center after negative charge injection. The dendrites are more easily injected in the single-point charge injection. There is a decay in the difference between the highest (lowest) potential point and the initial potential over time, and the rate of decay gradually decreases over time. When comparing the rate of decay of the potential change signal caused by the single-charge injection on the four curves with time, it is evident that the dendrites decay at a faster rate than the spherulites. When a single point of charge injection is carried out, the dendritic phase is more sensitive to charges. This is similar to the case when a regional charge injection is conducted.

Since the potential barrier between the metal-dielectric prevents charge injection into the dielectric, the degree of charge injection is mainly determined by the height of the potential barrier at the interface $\varphi \left(x_{max}\right)$ [31]

(2) $$\varphi \left(x_{max}\right)=\varphi_{m}-\chi -\sqrt{\frac{e^{3}E}{4\pi \varepsilon \varepsilon_{0}}}$$

where $\varphi_{m}$ is the metal work function, χ is the electron affinity energy of the dielectric surface, *e* is the charge quantity, *ε* is the relative permittivity of the dielectric film, *ε*_0_ is the permittivity of free space (8.85 × 10^−12^ F/m), and *E* is the constant electric field. The dendritic α phase has a lower intrinsic *ε*’. The dendritic α phase has a lower charge injection barrier when all else is equal. Thus, it is more susceptible to charge injection. In addition, the conductivity of the dendritic α phase is larger than that of the spherulitic β phase, which is beneficial to charge dissipation, so a faster potential decay rate accompanies it [32,33,34].

### 3.3. Microzonation Piezoelectric Properties of Films with Different Crystalline Phases

**Figure 7:**
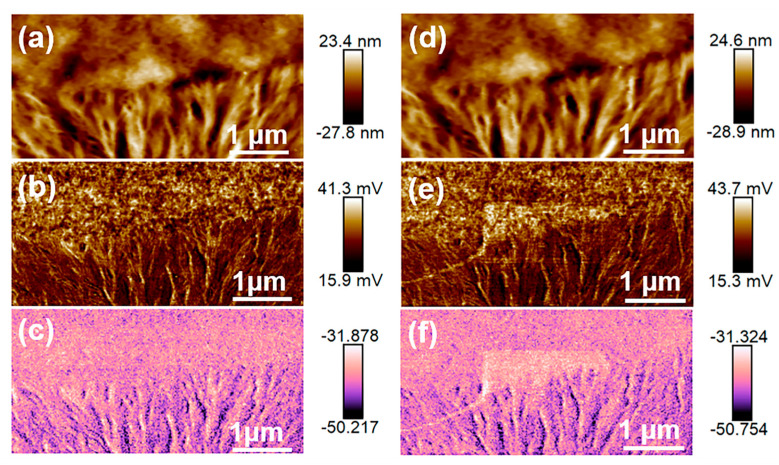
The piezoelectricity of PVDF film. (**a**–**c**) The height, amplitude, and phase images of the non-polarized PVDF film; (**d**–**f**) the height, amplitude, and phase images of the polarized PVDF film.

Figure 7 records the height, phase, and amplitude images of the non-polarized and polarized (±10 V) films with a +8 V driving voltage in the 5 × 2.5 µm^2^ region. The polarization condition in Figure 7d–f is polarization with −10 V in the central 4 × 2 μm^2^ area and then polarization with −10 V in the central 2 × 1 μm^2^ area. It can be seen from the height images that the film experiences no damage during the polarization process by the PFM contact mode. When the tip bias with opposite polarity is applied, a clear polarization reversal phenomenon occurs in the phase image of the spherulite, while the dendrite does not have that phenomenon. This also indicates that the spherulite is in the polar β phase and the dendrite is in the non-polar α phase. The −10 V polarization voltage has almost no effect on the polarization direction because PVDF exhibits the same spontaneous polarization direction as the polarization direction of the applied negative electric field. The amplitude image reflects the local piezoelectric response intensity. The larger the amplitude, the stronger the piezoelectric response in the area. A significantly enhanced piezoelectric amplitude can be seen with a +10 V polarization voltage. The micro-region *d*_33_ of PVDF film can be estimated by Equation (3) [35]:

(3) $$d_{33}=\frac{A\times deflection\;sensitivity}{V\times vertical\;deflection\;gain}$$

where *A* is the regional average amplitude, *V* is the AC driving voltage (+8 V), deflection sensitivity is 95.60 nm/V, and vertical deflection gain is a fixed value of 16. The *d*_33_ values for the non-polarized area, −10 V polarized area, and +10 V polarization area are calculated to be about 22.70, 23.75, and 26.59 pm/V, respectively. Compared with the non-polarized area, a bias induced the enhancement of ferroelectric polarization.

## 4. Conclusions

In this work, PVDF films with two typical crystalline phases (α and β) were prepared, and the single crystal charge behavior and piezoelectric properties of different crystalline phases of PVDF were directly investigated at the nanoscale using AFM. KPFM was employed to analyze the electrical response differences between the two crystals under charge injection, and it revealed that the dendritic α phase has a lower charge injection barrier, is more susceptible to charge injection, and has a faster charge dissipation rate. It is more vulnerable to charge accumulation at the interface between the α- and β-phases of PVDF, leading to increased interfacial polarization. In addition, PFM studies show that the amplitude and phase diagrams of the α-phase are unchanged under ±10 V bias, while the spherical crystalline β-phase undergoes a polarization-reversible transition and a significant increase in piezoelectric amplitude, which is consistent with its polar intrinsic properties. It is demonstrated that the crystal structure of PVDF has a great influence on charge injection, accumulation, and transport.

## Data Availability

The raw data supporting the conclusions of this article will be made available by the authors on request.

## Supplementary Materials

The following supporting information can be downloaded at: https://www.mdpi.com/article/10.3390/nano14191555/s1, Figure S1: (a) height (the red dot represents the charge injection site) and (b−h) Δ*V*_SP1_ images of the single-point positive charge injection region of the dendrite; Figure S2: (a) height (the red triangle represents the charge injection site) and (b−h) Δ*V*_SP1_ images of the single-point positive charge injection region of the spherulite; Figure S2: (a) height (the red triangle represents the charge injection site) and (b−h) Δ*V*_SP1_ images of the single-point negative charge injection region of the spherulite.
