# Supplementary material for: Electrical Response of Different Crystalline Microregions in Poly(vinylidene fluoride)
Source: Nanomaterials (Basel). 2024 Sep 26;14(19):1555. doi: 10.3390/nano14191555 (PMC11478445; doi:10.3390/nano14191555)
Supplement: Supplementary file 1 [file nanomaterials-14-01555-s001.zip › nanomaterials-3201575 - supplementary.pdf]

SUPPORTING INFORMATION

Electrical Response of Different Crystalline Microregions in Poly(vinylidene fluoride)

Mengyue Su, Jun Zhou *, Yuqing Chen, Yilong Wang, Gan Jin, Haiyang Wang, Jiacheng Zhou, Xiaoyue Pang, Zepeng Lv and Kai Wu *

Center of Nanomaterials for Renewable Energy, State Key Laboratory of Electrical Insulation and Power Equipment, Xi'an Jiaotong University, Xi'an 710049, China
* Correspondence: zhoujun@mail.xjtu.edu.cn (J.Z.); wukai@mail.xjtu.edu.cn (K.W.)

-10 V Bias Single-point Charge Injection to Dendritic α Phase

The -10 V bias charge injection was selected at the dendrite center, and its initial surface potential distribution was relatively uniform. Figure S2c-h shows the images of the potential difference obtained by scanning after 10 min of single-point negative charge injection. A blue area with a red edge appears in the center of the potential difference image after injecting a negative charge. The blue area with a red edge means that the potential here is lower. This indicates that the negative charges are successfully injected into the center point of the sample through the tip. In the subsequent potential difference images, the blue area in the center of the potential difference image changes to red, after which the red area fades in color.

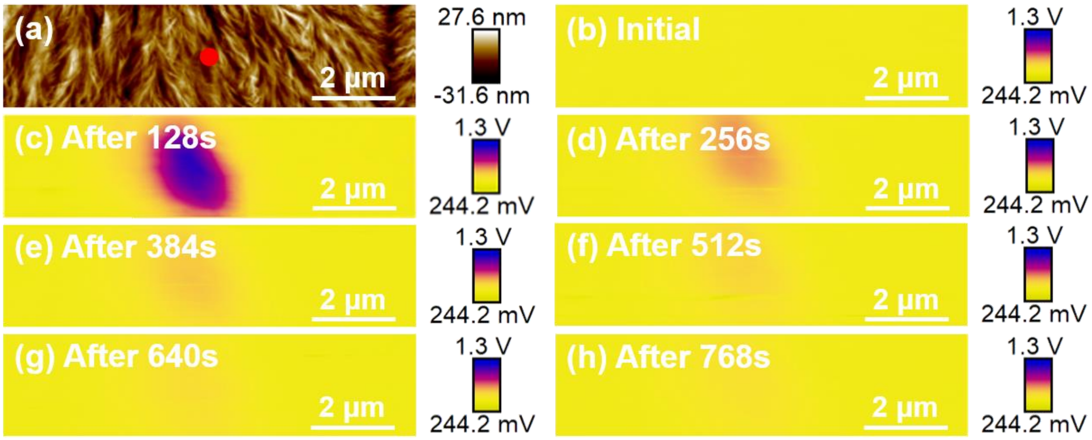

Figure S1. (a) height (the red dot represents the charge injection site) and (b–h) ΔV_{SP1} images of the single-point positive charge injection region of the dendrite.

+10 V Bias Single-point Charge Injection to Spherulitic β Phase

The +10 V bias charge injection was selected at the spherulite center, and its initial surface potential distribution was relatively uniform. A brighter area appears in the center of the potential difference image after injecting a positive charge. The brighter area means that the potential here is higher. This indicates that the positive charges are successfully injected into the center point of the sample through the tip. The brighter area at the center gradually becomes shallower in the subsequent potential difference images.

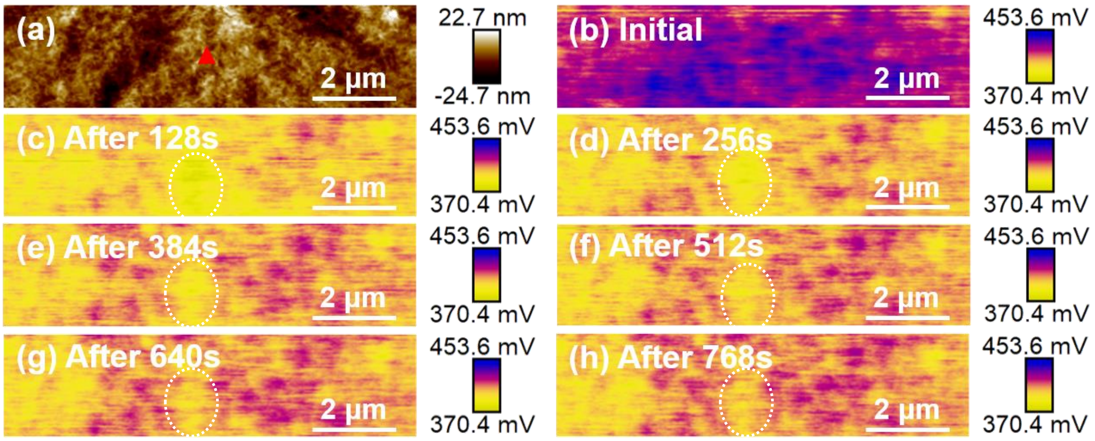

Figure S2. (a) height (the red triangle represents the charge injection site) and (b–h) ΔV_{SP1} images of the single-point positive charge injection region of the spherulite.

-10 V Bias Single-point Charge Injection to Spherulitic β Phase

The -10 V bias charge injection was selected at the spherulite center, and its initial surface potential distribution was relatively uniform. A blue area with a red edge appears in the center of the potential difference image after injecting a negative charge. The blue area with a red edge means that the potential here is lower. This indicates that the negative charges are successfully injected into the center point of the sample through the tip. In the subsequent potential difference images, The blue area in the center of the potential difference image changes to red, and then the red area fades.

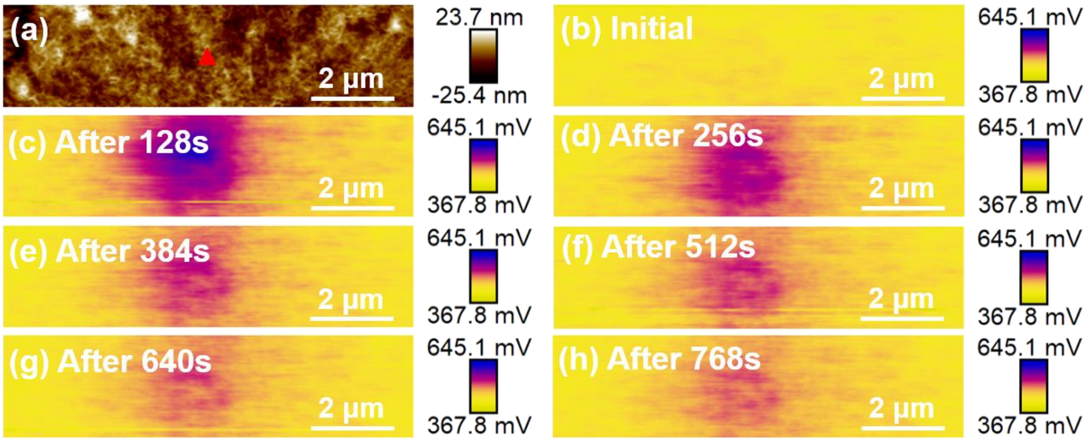

Figure S3. (a) height (the red triangle represents the charge injection site) and (b–h) ΔV_{SP1} images of the single-point negative charge injection region of the spherulite.
